# Of the Disposition Made of Communications with Reference to Improvements Made by the Authors

**Published:** 1864-03

**Authors:** B. Wood

**Affiliations:** Albany, N. Y.


### OF THE DISPOSITION MADE OF COMMUNICATIONS WITH REFERENCE TO IMPROVEMENTS MADE BY THE AUTHORS.

BY B. WOOD, M. D.

In your January number it was proposed to speak as to the exclusion from professional journals, of communications relating to improvements made by the authors and offered for sale.

Although the principle of adjudication referred to should, upon examination prove faulty and inexpedient for general adoption, it is proper to premise that I do not undertake to show this for vindication of a hearing through the journals in regard to anything made by me, for all this will find place in another form. Nor will I be charged with having *obtruded* upon our journals, especially upon one whose rule of adjudication is under consideration. The particular instance is in itself nothing, the disposition made of that commuica-tion was of no moment whatever, and only becomes so as involving a principle.

An independent mind, or a reckless one, a man self-confident, or supported by stout backers, even the timid and irresolute made bold and resolute from necessity, if denied through one medium will likely appeal to another. But the great majority need cheer and encouragement. Many will falter at the threshold whose talents fit them for great usefullness.

A public journal is a potent thing. The editorial position is, or ought to be a high one; often excites a degree of reverence, even awe, from whose decision few care to appeal. None are, perhaps, but who possess a certain sensibility which shrinks from observation in rebuff, content to submit to any adjudication rather than give occasion for whisperings. If it be in conformity to a rule of journalism, what use to appeal? And what avail to confront such formidable odds? Journals have great and perpetual influence; they should use it well and wisely. They are the arbiters. They are the promulgators, but others are workers. Let not the work of progress be impeded by partial and inexpedient rules of arbitration and promulgation.

For reasons intimated, although the occasion which has enlisted this discusion be utterly inconsequential and lighter than thin air, yet the principle involved is consequential and of weight.

A clear understanding requires a full quotation of the statement involving the principle at issue. I supply the elipses in brackets.

"There are many communications received [by the Dental Cosmos] in reference to improvements made by the authors,[of such communications] which [communications] are important to the profession, but which are usually declined as communications, [though not as advertisements ?] when they [the improvements] are offered for sale to the profession. This rule applies to myself as well as to other parties."

Almost every improvement is offered for sale to somebody, even improvement in skill is offered for sale *i. e.*, at a price to our patients. It is not then intended to debar information in regard to things because offered for sale. If offered for sale "to the profession," they come within the rule. In such cases communications in reference to them are certainly "important to the profession;" and one would naturally suppose a journal devoted to its interests would be prompt to convey information in relation thereto. But most improvements with which professional journals have to do come under this class; all the different materials, apparatus, fixtures, instruments, etc. There is much commuicated through the journals, the Cosmos as well as others, in reference to these things, their composition, preparation, construction, uses, the occasions and modes of employment, &c. The discrimination appears strictly confined to those who make improvements. *They* are excluded from a hearing, and the profession from hearing *them* in reference to their own improvements when these are offered for sale. The idea probably is that they are less competent or trustworthy than others, on such matters; yet I can not perceive what safe guard this rule affords. Those who are debarred may get others to write or father their communications; and it is surely more honorable for one to speak directly for himself, under his own hand, besides that, he is then under wholesome restraint, being under close scrutiny and held accountable for what he says.

This rule or custom appears to be of recent adoption. I was altogether unaware of it until receipt of the notification thereof, dated Sept. 30, 1863. The files of the Cosmos show many communications in reference to improvements made by the authors, and offered for sale to the profession; as witness those in reference to contiuous gum work, sponge gold, amalgum, and other material; improved instruments and apparatus, new materials and processes for filling and mounting teeth, the different improvements in gutta percha, rubber, vulcanite, &c., with the various details as to their advantages, uses, manner and methods of employment, &c.,

&c ; all which materials, apparatus and articles are offered for sale by the proprietor of the Cosmos, and advertised by him through that medium. Not to enumerate, we may instance the attention bestowed upon a single article, Oxichloride of Zinc, under its various names, which article is offered for sale, and which I judge from the editorials in the Cosmos in regard to it, is considered an improvement by that journal. I do not count the communications of others than the makers, though they equally served as an advertisement, if that be the ground of objection. And certainly the communications from those who manufacture the article, although confined mostly to its advantages, were not less important or instructive than those of others, not even excepting the very many and able EDITORIALS in reference to it.

Reviewing the new edition of Harris' Principles and Practice of Dental Surgery, *Cosmos*, October, 1863, Prof. McQuillen, referring to this article, (and probably unaware of the above rule,) makes the following remarks:

"It is somewhat surprising that, in describing the material used for filling teeth, no mention is made of *Osteo-Dentine.* The omission was no doubt unintentional, for even if the gentlemen who had charge of this department does not regard the article favorably, the fact of its being used as it is, very largely by the profession, demands of necessity that decided attention should be directed to its composition, employment, and the various arguments for or against its use."

The course here indicated seems to be the proper one to pursue in reference to all alledged improvements in use, until their nature and qualities be fully understood.

But why this discrimination against the makers of improvements offered for sale? Are they less reliable, watched as they are at every point, than others interested, perhaps, as much in respect to articles they are *using*, and have become proficient with? Are others wholly free from self-interest, favoritism and prejudice? Is it more detrimental to the profession to have an *improvement overrated*, if that is the fear, than *underrated?*

It is to be presumed men will be well informed in relation to their own improvements and collateral subjects. The information in their possession is apt to be precise and definite, and therefore of peculiar service to earnest enquirers. There are peculiar considerations to restrain them in the preparation and presentation of communications for publicity, which others do not feel and, perhaps, can not understand. Incredulity, prejudice, the liability to imputation, the hesitancy with which the facts are accepted, and the security with which they are scanned, render it no grateful task. And as readers are guardful and disposed to make broad allowance for what is said commendatory, there is little danger of being unduly influenced by this class of writers. Accordingly while their communications may be more complete and accurate in point of information, they subserve the purpose of an "advertisement" far less than if written by others.

What should be classed with "advertisements," and what with "communications," in a publication, might perplex one to define. But the announcement of any information important to the profession is doubtless due a place once, as a communication, whether it benefits the person making it or not. After that, if the writer, or any one else, desires to keep it before the profession for his own or another's benefit, or other purposes, he might properly be referred to the advertising sheet; since one statement of the same information is all he can ask, and all that a journal promises to give, unless it be for elucidation of new matter. It is on record and easily referred to. The advertising sheet is no record, being destroyed in the binding.

A case at hand serves to illustrate these points. In the Dental Cosmos from February to August, 1862, appeared a series of communications from the editor, Dr. J. D. White, describing his improved plugging forceps, representing their form and construction, by cuts, the manner of using them, and their advantages over the complicated and worthless forms in use till then. "Ours," he says, "are seven in number and very simple," etc. "We have promised Dr. S. S. White, if he will get them up properly, that we will *inspect* them before they go out to the profession, so that the full value of them may, perhaps, be better understood; as every thing depends upon their nice construction, which we can not describe."

In the advertising sheet of the number for July, 1862, the proprietor of the Cosmos, Dr. S. S. White, announces the improvement for sale by him as follows:

"As many forceps have been sold purporting to be made according to Dr. J. D. White's patterns, which have not been correct in shape, and some of them so badly constructed as to be entirely useless, we have undertaken to have them manufactured, and ensure their correctness, they will be submitted to Dr. White's inspection before they are offered for sale. The sets consist of seven forceps, at $1,25 each." Here follow the cuts with brief description, quoted from Dr. J. D. White's communications.

Here we perceive a clear distinction between a communication and an advertisement. And, although this case would seem to be an infraction of the rule adopted by the Cosmos, yet who will say that Dr. J. D. White had not a right to a place for his communications with reference to his own improvement upon the records of the journal, notwithstanding said improvement, was offered for sale to the profession, no matter whether for his own benefit or that of another, provided the matter was of importance to the profession. And who would interpose objection to the profession's having his own description of it, rather than that of others, who might be as much at fault in the description, as those who undertook to manufacture the article without his inspection, proved to be in the construction?

A publisher having a third of his journal set apart expressly for advertising what he has for sale, while a goodly portion of the other two thirds is, of necessity, tributary thereto, may properly adopt rules for his own government, which would prove quite inexpedient if applied to correspondents.

We have assumed that the communications *are* important to the profession, and also that the journal *is* devoted to the interests of the profession. Admitting these two propositions, the subject hardly requires serious discussion. Any rule or usage of the nature referred to is so transparently untenable and impracticable, and its general adoption would be so obviously mischievous, that argument is unnecessary. If it were a received rule in dental journalism it would tend to reverse the wheels of progress and drive our art back to the place of starting. It would exclude all precise knowledge of new improvements, bar the door against information from the most direct and competent sources, and exclude the class of thinkers and doers, to whom we look for available instruction, while leaving the channel wide for the dissemination of errors and crudities at the hands of cynics, critics, and superficial observers. Inaugurated by one of our popular journals, it seemed fit that the false prinple involved in its inception should be noticed, for there are improvements continually originating in reference to which it would be desirable to hear from the originators, through the customary channels. For one, I should be much gratified to peruse some of the many important communications which the Cosmos has consigned to the "dark corner;" and I trust it may yet be induced to publish them itself or submit them to its contemporaries for publication.

ALBANY, N. Y.

Since the above, I find in a late number of the Dental

Cosmos, in an Editorial on Oxichloride of Zinc, the following remark, in regard to announcements concerning it:

"It is proper and right, perhaps the duty, for every inventor to put his views and productions forth as fast as he can in everything that looks towards benefitting mankind."

This would seem to leave nothing undetermined except as to the form or manner of putting forth.

